# Culture-based studies of intestinal lactobacilli in young people and centenarians

**DOI:** 10.3389/fmicb.2026.1746411

**Published:** 2026-03-10

**Authors:** Imbi Smidt, Tiiu Roop, Reet Mandar, Jelena Stsepetova, Siiri Koljalg, Kalle Kilk, Indrek Soidla, Mare Ainsaar, Helgi Kolk, Epp Sepp

**Affiliations:** 1 Department of Microbiology, Faculty of Medicine, Institute of Biomedicine and Translational Medicine, University of Tartu, Tartu, Estonia; 2 Department of Biochemistry, Faculty of Medicine, Institute of Biomedicine and Translational Medicine, University of Tartu, Tartu, Estonia; 3 Faculty of Social Sciences, Institute of Social Studies, University of Tartu, Tartu, Estonia; 4 Department of Internal Medicine, Institute of Clinical Medicine, University of Tartu, Tartu, Estonia

**Keywords:** biochemical profile, centenarians, lactobacilli, metabolic activities, microbiota, probiotics

## Abstract

**Introduction:**

Biological ageing is associated with physiological changes, including alterations in the gut microbiota. Lactobacilli may contribute to host health and longevity, yet their composition and functional properties in centenarians remain poorly characterized. The present study aimed to compare cultured intestinal lactobacilli from centenarians and young adults and to identify strains with potential probiotic properties.

**Methods:**

Fecal samples were obtained from centenarians (*n* = 25) and young adults (*n* = 25). Lactobacilli were isolated using culture-based methods and identified to the species level. Antibiotic susceptibility testing was performed for all isolates. Biochemical and metabolic properties of antibiotic-sensitive strains were determined.

**Results:**

Twenty *Lactobacillus* species were identified. Six species were shared between groups, 12 were unique to centenarians, and two to young adults. Although overall Lactobacillaceae abundance was similar, centenarians showed greater species richness and a higher relative proportion of lactobacilli. Isolates from centenarians exhibited distinct carbohydrate fermentation patterns and metabolic profiles, including higher levels of acylcarnitines, arachidonic acid, and selected bile acids.

**Discussion:**

Lactobacilli isolated from centenarian demonstrate distinct compositional and metabolic characteristics compared with those from young adults. These differences may reflect functional adaptations potentially relevant to healthy ageing and could inform the selection of candidate strains for future probiotic development.

## 1 Introduction

The human gut microbiota harbors trillions of bacteria that play important roles in health, disease, and aging. Biological aging is a multifactorial process involving all human physiological processes, including the gut microbiota (O'Toole and Jeffery, 2018). The gut microbial diversity generally decreases with age (Lynch and Pedersen, 2016), which is likely due to changes in physiology, diet, medication, and lifestyles. In addition to decreased diversity, the changes in the gut microbiota composition correlate with frailty, inflammation, and neurodegenerative disorders in the elderly (Santoro et al., 2018; Johnson, 2020; Xu et al., 2021).

However, centenarians have been used as a model of healthy aging because of their ability to delay or avoid chronic diseases (Kong et al., 2016; Wu et al., 2019). We previously compared the gut microbiota of centenarians and young people (Sepp et al., 2022). Similar to previous Italian, Indian, Japanese, and Chinese studies, centenarians had a higher richness and diversity of gut microbiota than young people in these countries (Kong et al., 2016; Santoro et al., 2018; Wang et al., 2022).

The composition of the microbiota is related to eating habits (Bourdeau-Julien et al., 2023). Our previous study showed that centenarians eat more potatoes and cereal products that contain large amounts of starch, fiber, and polyphenols (Sepp et al., 2022). These substances have a beneficial effect on the gut microbiota and their metabolites (Bibi et al., 2019). At the beginning of the last century, Metchnikoff proposed the hypothesis that eating fermented dairy products might affect life expectancy positively (Metschnikoff, 1908). Fermented dairy products contain large numbers of lactic acid-producing lactobacilli.

The genus *Lactobacillus*, together with related genera, comprises approximately 300 species that are extremely diverse at the phenotypic, ecological, genotypic, and metabolic levels. Currently, lactobacilli are divided into 25 genera, including the emended genus *Lactobacillus*, which includes host-adapted organisms referred to as the *Lactobacillus delbrueckii* group, *Paralactobacillus*, and 23 novel genera (Zheng et al., 2020). The generic term ‘lactobacilli’ will remain useful to designate all organisms that were classified as *Lactobacillaceae* until 2020 (Zheng et al., 2020). Lactobacilli are divided according to their habitat or ecological niche into vertebrate host-adapted, insect-adapted, nomadic, environmental, or free-living and unassigned strains (Zheng et al., 2020). According to the type of sugar fermentation, lactobacilli can be divided into three groups: homofermentative (OHOL), heterofermentative (FHEL), and obligate heterofermentative (OHEL) lactic acid bacteria (Mikelsaar et al., 2002, 2016).

Lactobacilli are frequently used in the development of probiotics. Probiotics improve the host's health by having antimicrobial effects, modulating immunity, and maintaining healthy microbiota within the host's gut, thereby promoting healthy aging (Sepp et al., 2018; Tsai et al., 2021). Previous reports indicated the role of lactobacilli in healthy aging and longevity. Lactobacilli isolated from food extend the life of the soil nematode *Caenorhabditis elegans* (Ikeda et al., 2007; Sharma et al., 2019). In an aging mouse model, *Lactobacillus casei* and the dietary fiber complex alleviated age-related cognitive impairment and protected brain and gut function (Ren et al., 2022). Lactobacilli isolated from centenarians affected the intestinal microbiota, reduced inflammation, and improved impaired spatial memory and motor dysfunction in aging mice (Fang et al., 2021).

We thus hypothesize that lactobacilli in the gut influence healthy gut microbiota and promote healthy aging. This knowledge would provide the opportunity to use probiotic lactobacilli for the modulation of gut microbiota and the prevention of aging (Deng et al., 2019). Unfortunately, we still know very little about centenarians' microbiota.

The aim of this study was to compare the cultured gut microbiota and microbiota of centenarians and young people and to find suitable strains for the development of potential probiotics.

## 2 Materials and methods

### 2.1 Study population

Young people (*n* = 25; age range 19–23 years, age median 21) were born in the late 1990s, and very old people were referred to as the centenarian group (*n* = 25; age range 96–103 years, age median 99). Centenarians were selected according to their preserved cognitive function. The clinical data of the subjects and more detailed inclusion criteria have been described previously by Sepp et al. (2022). The study was approved by the ethics committee of the University of Tartu (275/T-13).

Fecal samples were collected from all subjects and stored in domestic refrigerators at −20 °C until transportation to the laboratory. The samples were frozen at −80 °C until culture-based analyses for determining gut microbiota.

### 2.2 Cultural analyses

Weighted fecal samples were serially diluted (10^−2^ to 10^−9^) in pre-reduced phosphate buffer (pH 7.2) in an anaerobic glove box (Whitley A35 Anaerobic Workstation; UK) with a gas mixture consisting of CO_2_, H_2_, and N_2_ (5%; 5%; 90%). A quantitative analysis of the gut microorganisms was performed by culturing the serial dilutions on six agar plates: de Man Rogosa Sharpe Agar (MRS; Oxoid) and Rogosa Agar (Oxoid) for lactobacilli and streptococci, Fastidious Anaerobe Agar (FAA; Lab M) for anaerobes, Brazier's Cycloserine Cefoxitin Egg Yolk Agar (CCEY; Lab M) for *Clostridioides difficile*, Brilliance™ *E. coli*/coliform Selective Agar (Oxoid, Basingstoke, United Kingdom) for enterobacteria, and Saboraud Agar (Lab M) for yeasts. The Brilliance™ *E. coli*/coliform Selective Agar was incubated aerobically at 37 °C and inspected after 24 and 48 h. Sabouraud agar was incubated aerobically at 25 °C and inspected after 1 and 2 weeks. The MRS medium was incubated in a microaerobic atmosphere [SANYO CO_2_ incubator, MCO-19AIC(UV), Osaka, Japan] with a gas mixture of 10% CO_2_ for 2 days. Rogosa Agar, FAA, and Brazier CCEY were incubated in an anaerobic glove box for 2–4 days. The colony counts of the different fecal dilutions were recorded, and from the highest dilutions with growth, all colonies of different morphologies were analyzed for identification. Microorganisms were identified by matrix-assisted laser desorption/ionization time-of-flight (MALDI-TOF; Bruker, Bremen, Germany) at the genus level. Microbial counts were expressed in log colony-forming units per gram of feces (CFU/g). The detection limit of microorganisms was 3.0 log CFU/g. For each subject, the counts of different bacterial groups were calculated and summarized to obtain the total count of the cultivated intestinal bacteria. The relative share (%) of each bacterial group was calculated from the total counts.

### 2.3 Molecular identification of lactobacilli

The total DNA of *Lactobacillus* sp. was extracted using a QIAamp DNA Mini Kit (Qiagen, Germany) following the manufacturer's protocol for gram-positive bacteria. For identification of *Lactobacillus* sp., two 16S rRNA primers, CO1 (5′-AGTTTGATCCTGGCTCAG-3′) and CO2 (5-TACCTTGTTACGACT-3′), were used to generate an approximately 1.5 kb 16S rRNA product under PCR conditions described previously (Simpson et al., 2003).

Sequencing was carried out at the Institute of Genomics, University of Tartu, by the dideoxy method (Sanger et al., 1977) using a PRISM BigDye Terminator Cycle Sequencing Ready Reaction kit (Applied Biosystems Inc., CA, USA) in combination with an Applied Biosystems model 3730XL automated sequencing system.

Similarity searches for 16S rRNA gene sequences were performed in the GenBank database using the BLAST algorithm. Strains that have similarity values of 98–100% are considered to belong to the same species. The nucleotide sequences for the 16S rRNA gene were deposited with GenBank under accession numbers OL434975-OL435034 and OL435036-OL435052 (*n* = 77) for *Lactobacillus* sp.

### 2.4 Antibiotic susceptibility testing

The susceptibility of lactobacilli was assayed using E-test methods according to the European Food Safety Authority guidelines. The minimum inhibitory concentrations (MICs) of the following antimicrobials were determined: ampicillin, vancomycin, gentamicin, kanamycin, erythromycin, tetracycline, clindamycin, chloramphenicol, and streptomycin.

### 2.5 Detection of biochemical profile

The API 50 CH identification system (bioMérieux, Inc., Marcy l'Etoile, France) was used according to the manufacturer's instructions. The system enabled the detection of 49 biochemical parameters.

### 2.6 Metabolomic characterization

The metabolites secreted by lactobacilli to growth media were measured by using three protocols: (1) the Biocrates MxP Quant 500 kit (Biocrates Life Sciences AG, Innsbruck, Austria), (2) an in-house protocol for vitamins (based on Waters Corporation application notes, Yang et al., 2022), and (3) an in-house protocol for short-chain fatty acids. All measurements were performed on a Waters ACQUITY ultraperformance liquid chromatography Xevo TQ-XS mass spectrometer. The short-chain fatty acids were derivatized with 2-nitrophenylhydrazine and 1-ethyl-3-(3-dimethylaminopropyl) carbodiimide for 60 min at room temperature before analyzing on an ACQUITY Premier BEH C18 1.7 μm, 2.1 x 100 mm column.

### 2.7 Statistical analysis

Statistical analyses were performed using the PAST statistical software packages. The data were compared using the Wilcoxon rank sum test and paired *t*-test according to the data distribution (number of lactobacilli species, counts of lactobacilli, and antibiotic MIC values). Colonization with different *Lactobacillus* species was compared using Fisher's exact test. The diversity of microbiota was described by the Shannon diversity index, metabolite concentrations were compared by Student's *t*-test, and effect sizes were calculated using Cohen's d. Statistical significance was set at a *P-*value of < 0.05.

## 3 Results

### 3.1 Microbiota of the gut

A total of 32 families belonging to four phyla were isolated using the cultural method (Supplementary Table S1). In the centenarian group, we isolated 111 bacterial species from 27 families, and in the young group, 87 bacterial species from 25 families. The median number of families (median: 7 vs. 7) and the median number of species (median: 11 vs. 11) per subject were similar between young people and the centenarian group. The richness and diversity of the cultured gut bacteria did not differ between the groups.

Young people were more colonized by the family *Bifidobacteriaceae* (84% vs. 52%; *p* = 0.032) and the species *Bifidobacterium longum* (72% vs. 28%; *p* = 0.004) as compared to centenarians. In addition, *Bacteroides ovatus* (32% vs. 72%; *p* = 0.01) and *Bacteroides faecis* (4% vs. 32%; *p* = 0.036) species were more frequently isolated in the young group. The counts and relative share of *Coriobacteriaceae* were higher and *Enterococcaceae* were lower in young people when compared to centenarians (Supplementary Table S1).

### 3.2 Lactobiota of gut

**Figure 1:**
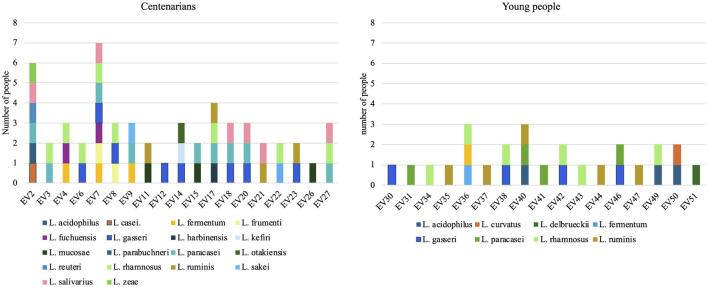
The colonization of centenarians (EV2-EV27) and young (EV30-EV51) people with different *Lactobacillus* species.

A total of 80 lactobacilli strains were isolated and identified: 54 from centenarians and 26 from young people. Colonization (80% vs. 72%) and *Lactobacillaceae* count were similar in centenarians and young people, but the relative share of lactobacilli was higher in centenarians (Supplementary Table S1). Centenarians had significantly higher species richness of lactobacilli as compared to young people (median 3 vs. 1 species; *p* < 0.001) (Figure 1).

Centenarians were more often colonized with the OHEL group (median 6 vs. 1 species; *p* < 0.05) and environmental lactobacilli (median 6 vs. 1 species; *p* < 0.05) than young people (Table 1). In addition, centenarians were more often colonized with *L. paracasei* (36%), *L. gasseri* (32%), and *L. rhamnosus* (32%), while young people were colonized with *L. rhamnosus* (24%) and *L. ruminis* (20%) (Figure 1, Table 1).

**Table 1:** Colonization of centenarians and young people with different *lactobacillus* species.

**Fermentation group**	**Species of lactobacilli**	**Young people *N* =**	**Centenarians *N* =**
FHEL	*Lacticaseibacillus casei*		1
	*Latilactobacilluscurvatus* ^*^	1	
	*Latilactobacillusfuchuensis* ^*^		2
	*Schleiferilactobacillusharbensis* ^*^		1
	*Lacticaseibacillus paracasei*	4	9
	*Limosilactobacillus reuteri*		1
	*Lacticaseibacillus rhamnosus*	6	8
	*Latilactobacillussakei* ^*^		1
	*Lacticaseibacillus zeae*		1
OHEL	*Limosilactobacillus fermentum*	1	3
	*Limosilactobacillus frumenti*		2
	*Lentilactobacilluskefiri* ^*^		1
	*Limosilactobacillus mucosae*		3
	*Lentilactobacillusparabuchneri* ^*^		1
	*Lactobacillus otakiensis* (*Lentilactobacillus otakiensis*)^*^		1
OHOL	*Lactobacillus acidophilus*	4	1
	*Lactobacillus delbrueckii*	1	
	*Lactobacillus gasseri*	4	8
	*Ligilactobacillus ruminis*	5	4
	*Ligilactobacillus salivarius*		6

FHEL, facultative hetero-fermentative; OHEL, obligate hetero-fermentative; OHOL, obligate homo-fermentative. ^*^Environmental species.

### 3.3 Antibiotic susceptibility of lactobacilli

The antibacterial susceptibility of the 77 isolated lactobacilli strains was determined according to the European Food Safety Authority guidelines (EFSA, 2012). As a result, 3.9% of the strains were resistant to ampicillin, 64.9% to kanamycin, 15.6% to streptomycin, 1.3% to gentamicin, 2.6% to erythromycin, 5.2% to tetracycline, 13% to clindamycin, 20.8% to chloramphenicol, and 19.5% to vancomycin, while 23.4% were sensitive to all tested antibiotics (Supplementary Table S2). Altogether, 21 strains were susceptible to all studied antibiotics; 12 of them belonged to the FHEL fermentation group, and nine were of nomadic habitat.

Of the 77 studied strains, 51 *Lactobacillus* strains were isolated from centenarians and 26 from young people (Supplementary Table S2). Centenarian lactobacilli strains had higher chloramphenicol MIC values compared to lactobacilli strains from young people (*p* = 0.01). According to the fermentation group, 33 lactobacilli strains belonged to the OHOL group, 33 strains to the FHEL group, and 11 to the OHEL group. The MIC values of ampicillin in the FHEL group were higher than those in the OHOL and OHEL groups (*p* = 0.007; *p* = 0.000007). The MIC values of tetracycline in the OHEL group were higher than those in the FHEL and OHOL groups of lactobacilli (*p* = 0.000004; *p* = 0.0000007). The MIC values of kanamycin of the OHOL group lactobacilli were higher compared to the FHEL and OHEL group strains (*p* = 0.04; *p* = 0.004). According to the habitat or ecological niche of lactobacilli strains, 39 strains were vertebrate as host-adapted, 30 were nomadic, 5 were environmental, and 3 were free-living strains (*L. fermentum*). The MIC values of chloramphenicol in nomadic strains were higher compared to environmental and vertebrate strains (*p* = 0.03; *p* = 0.007).

### 3.4 Biochemical profile of lactobacilli

The carbohydrate fermentation was determined on 18 strains of lactobacilli (13 from centenarians and 5 from young people) that were sensitive to different antibiotics according to ISO standards. A commercial kit (API 50 CHL) was used to determine the fermentation of carbohydrates (Supplementary Table S3). The fermentation of carbohydrates by *L. rhamnosus*, belonging to the FHEL group, was affected by the age of the person from whom the strain was isolated. *L. rhamnosus* strains isolated from centenarians fermented D-Arabinose (monosaccharide) and L-Fucose (monosaccharide), and strains isolated from young people fermented Methyl-αD-Glycopyranoside (monosaccharide), D-Maltose (disaccharide), and D-Turanose (disaccharide).

### 3.5 Metabolic activities of lactobacilli

**Figure 2:**
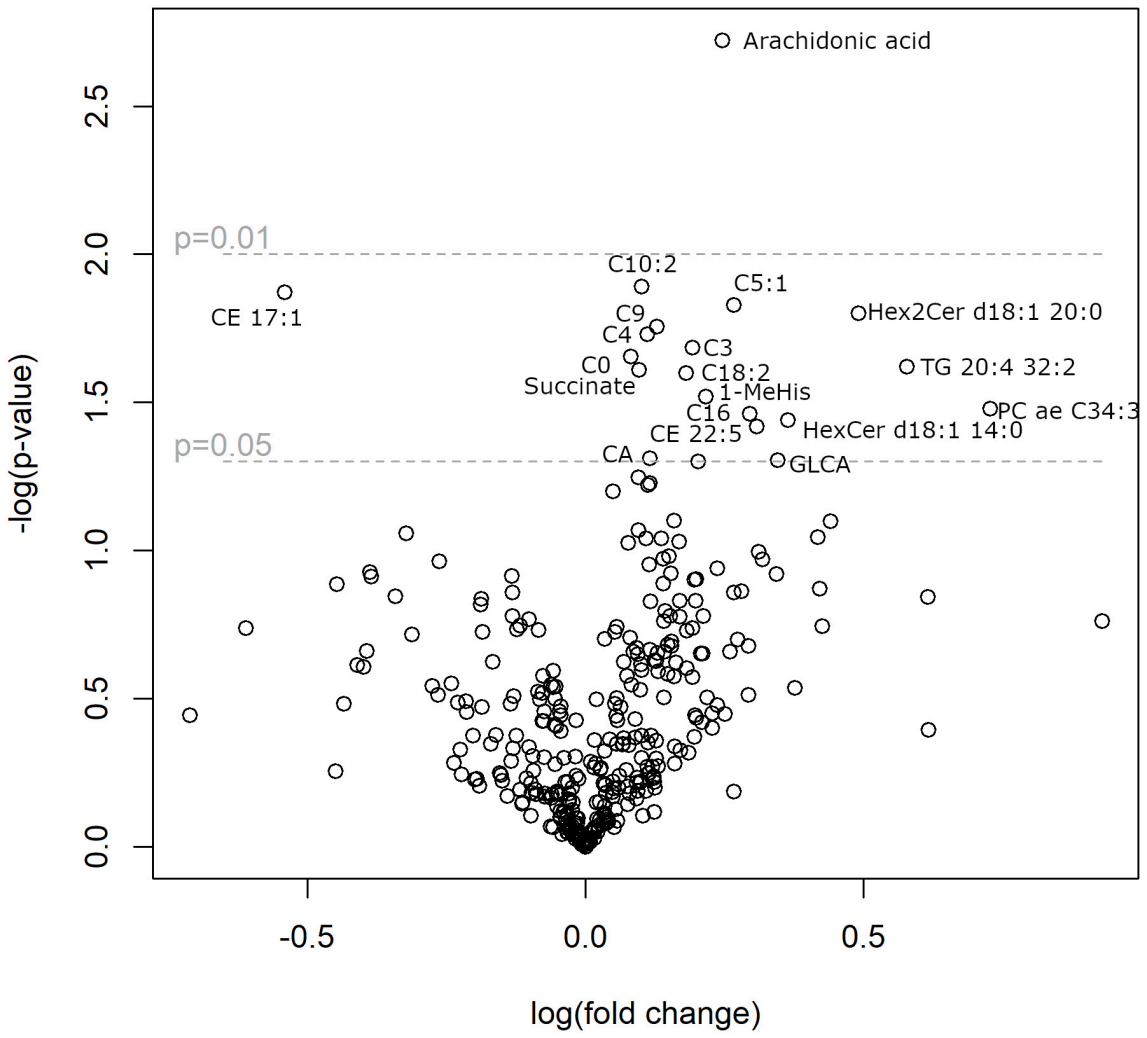
Fold change in culture media metabolite levels of centenarian/young strains (x-axis, logarithmic scale) vs. statistical significance of the change (y-axis, negative logarithmic scale). The most significant changes are annotated: C, carnitine esters with fatty acids with denoted length and number of double bonds; CE, cholesterol ester with fatty acids with denoted length and number of double bonds; HexCer, hexylceramide with a fatty acid with a denoted length and number of double bonds; PC ae, plasmalogen with a fatty acid with a denoted length and number of double bonds; TG, triglyceride with a fatty acid with a denoted length and number of double bonds; CA, cholic acid; GLCA, glycolitocholic acid.

The metabolic activities were determined on 18 strains of lactobacilli that were sensitive to different antibiotics. In the targeted approaches, more than 300 metabolites in the growth media were quantified (Supplementary Table S4). Comparison of centenarians' strains (*n* = 13) with young people's strains (*n* = 5) revealed significant differences in the levels of arachidonic acid, several acylcarnitines, and a few bile acids, ceramides, phosphatidylcholines, and cholesterol esters (Figure 2). In addition to lipids, succinate and 1-methyl-histidine were produced at higher levels by the strains from centenarians.

To determine whether the differences in metabolism could stem from the fermentation group and ecological habitation type, an analysis of variance between these categories was performed. Succinate, triglyceride, 1-methylhistidine, and cholic acid also differed between the fermentation groups and ecological niches. Glycolithocholic acid levels varied among the fermentation groups. Palmitoylcarnitine (C16) and ceramide with a C20:0 fatty acid residue are associated with the ecological origin of the strain. The other metabolites annotated in Figure 2 were not associated with either fermentation type or ecological origin.

## 4 Discussion and conclusion

Microbiome research has dominated the field of gut microbiota over the last two decades. However, we must not forget the “old” cultural methodologies that allow us to isolate different microbes and determine their various properties. Each of these approaches has advantages and drawbacks that can be overcome if used synergistically. We used the same material as in our previous microbiome study, applying next-generation sequencing (Sepp et al., 2022). In the present study, we applied quantitative cultures in order to compare the culturable gut microbiota of centenarians selected based on preserved cognitive function and young people. In addition, this approach allowed us to identify antibiotic-sensitive strains of lactobacilli that could be used in the future for the development of novel probiotics.

The microbiota of centenarians had more culturable microbial species, including lactobacilli, compared to those of young people. At the same time, young people were more often colonized by bifidobacteria (*B. longum*) and bacteroids (*B. ovatus* and *B. faecis*). They also contained high amounts of *Coriobacteriaceae*. Centenarians had an increased proportion of lactic acid-producing enterococci and lactobacilli compared to young people.

Our previous molecular studies (Sepp et al., 2022) did not coincide with the results of the present cultural study. This may be due to the differences in non-culturable bacteria between the young and centenarians. Using molecular methodology, the abundance of *B. longum* was higher in centenarians, but in the present study, young people were more colonized with the aforementioned bacteria. In addition, young people had an increased number of *Coribacteriaceae*. Kim and co-authors compared the fecal microbiota of overweight or obese adults in two groups—a metabolically healthy group without any components of metabolic syndrome and a metabolically unhealthy group. They found that *Coriobacteriaceae* was associated with good metabolic health in the overweight and obese populations (Kim et al., 2020). Among healthy young adults, the gut “R*uminococcaceae*- and *Coriobacteriaceae*-dominant community” was positively associated with good cognitive performance (Oluwagbemigun et al., 2022). An increased abundance of lactobacilli has been previously reported in Asian studies. Chinese centenarians had an increased proportion of lactobacilli compared to young adults and obese people (Wu et al., 2019). Lactobacilli were more common in the subjects of longevity villages than in the elderly and adults in urbanized towns (Kim et al., 2019).

Lactobacilli are one of the most important bacterial groups in humans, and their number and composition play a vital role in maintaining the health of the intestinal tract and improving the body's immune system (Mikelsaar et al., 2002; Rastogi and Singh, 2022). The colonization of different species of lactobacilli depends on the living environment, diet, and age (Ahrne et al., 2005; Stsepetova et al., 2011; Drago et al., 2012; Mikelsaar et al., 2016; Pan et al., 2016; Davoodabadi et al., 2020). In our study, the microbiota of centenarians were species rich compared to those of young people. In total, 20 different lactobacilli species were isolated. Only six species (*L. paracasei, L. rhamnosus, L. fermentum, L. acidophilus, L gasseri, L. ruminis*) were found in both centenarians and young people, whereas twelve species were found only in centenarians (*L. casei, L. fuchuensis, S. harbensis, L. reuteri, L. sakei, L. zeae, L. frumenti, L. kefiri, L. mucosae, L. parabuchneri, L. otakiensis, L. salivarius*) and two species (*L. curvatus, L. delbrueckii*) only in young people. *L. sakei* and *L. casei* were found in both Italian centenarians and younger adults, whereas three species (*L. reuteri, L. johnsonii, L. rhamnosus*) were found only in centenarians, and four species (*L. fermentum, L. plantarum, L. paralimentarius, L. gasseri*) were found in younger adults (Drago et al., 2012). Hence, the results of the Estonian and Italian studies do not coincide in that aspect. Thus, longevity cannot be achieved with a specific species of lactobacilli; however, the fermentation group of lactobacilli can be important. Homofermentative and heterofermentative lactobacilli produce different products from fermented carbohydrates. OHOL group lactobacilli mainly produce lactic acid from sugars, whereas heterofermentative groups such as FHEL and OHEL produce lactic and acetic acids, alcohol, and carbon dioxide. In addition to the substances listed above, the metabolic products of the OHEL group include various other acids, such as succinate. Centenarians had more OHEL group lactobacilli compared to young people. Metabolic products of the OHEL group are important for other gastrointestinal bacteria for cross-feeding. Drinking the probiotic kefir containing *L. fermentum* ME-3 (belonging to the OHEL group) increased the diversity of the intestinal microbiota, particularly the richness and diversity of the microbiota (Sepp et al., 2018). *L. fermentum* belongs to the nomadic group; human colonization occurs mostly with fermented foods or probiotic products (Naghmouchi et al., 2020; Zheng et al., 2020; Adesulu-Dahunsi et al., 2022).

To develop novel probiotics, it is necessary to use non-pathogenic bacterial strains. The lactic acid bacteria, bifidobacteria, and lactobacilli are mostly non-pathogenic and are widely used in the food industry. Currently, one crucial issue is antibiotic resistance and its spread (Collaborators, 2022, 2024). Within the framework of One Health, various measures have been proposed to prevent the spread of antibiotic-resistant bacteria among humans, animals, and the environment, including food (Velazquez-Meza et al., 2022). The presence of resistant bacteria in the human food supply chain has been documented, which presents a potential exposure route and risk to public health (Campedelli et al., 2019; Bennani et al., 2020; Anisimova et al., 2022). Antibiotic-resistant probiotic strains can spread their resistance genes to other human bacteria, thereby promoting the spread of resistance and treatment failure in patients (Nawaz et al., 2011). In the present study, over half of the lactobacilli strains were resistant to kanamycin, and one-fourth were resistant to clindamycin, chloramphenicol, and vancomycin. Lactobacilli are usually resistant to aminoglycosides such as kanamycin; this resistance is considered intrinsic and originates from the low impermeability of the lactobacillar cell surface for aminoglycosides (Anisimova and Yarullina, 2019). Lactobacilli isolated from centenarians and belonging to a nomadic group were more resistant to chloramphenicol compared to strains of young people and strains from other origins. The FHEL group lactobacilli strains were more resistant to ampicillin, the OHEL strains to tetracycline, and the OHOL strains to kanamycin compared to other fermentation groups. Almost one-fourth of the *Lactobacillus* strains were sensitive to all the antibiotics we determined. Among the antibiotic-susceptible *Lactobacillus* strains, two-thirds belonged to the FHEL fermentation group, and half were from a nomadic habitat.

In addition to the antibiotic resistance pattern, during the development of new probiotics, it is necessary to know the strain-specific properties of strains that positively affect human health. We determined the strain-specific properties (pattern of metabolic products) of antibiotic-sensitive lactobacilli, on the basis of which it will be possible to select new probiotic candidates with specific properties in the future.

The strains of *Lacticaseibacillus rhamnosus* isolated from centenarians and young people had different fermentation of carbohydrates, which may be related to dietary habits. Strains isolated from centenarians fermented monosaccharides such as arabinose and fucose. L-arabinose is found in certain plant cell walls, including many grains and plant gums. It has half the sweetness of sucrose (Ahmed et al., 2022). Studies examining the consumption of L-arabinose in humans showed that it reduced glycemic and insulinemic responses (Pol et al., 2022). Fucose is present in a variety of glycolipids and glycoproteins produced by mammalian cells (Werz et al., 2007). It is continuously synthesized by humans in the form of fucosylated glycans and fucosyl-oligosaccharides and is delivered into the gut. Gut microorganisms metabolize L-fucose and produce short-chain fatty acids, which are absorbed by epithelial cells and used as energy sources or signaling molecules to promote human health (Kim et al., 2023). For example, *Lacticaseibacillus rhamnosus* GG, a leader in the probiotic food market, metabolizes fucose in the intestine (Cheong et al., 2022). *L. rhamnosus* strains isolated from young people fermented methyl-αD-glycopyranoside and disaccharides such as maltose and turanose. Maltose, or malt sugar, is produced from the starch in the grains during the germination of barley grains. Turanose is found naturally in plants and honey. This sugar has a low glycemic index and can be used as a sweetener in low-calorie foods. The digestibility of turanose by intestinal enzymes is lower when compared to maltose (Ponnurangam and Balaji, 2024). The strain-specific properties of *L. rhamnosus* may be related to the dietary habits of centenarians and young adults, i.e., the strains ferment the sugars that people eat. Centenarians consume more potatoes, and their lactobacilli ferment arabinose (Sepp et al., 2022). Potato lectin is an unusual glycoprotein containing approximately 50% carbohydrates by weight, and of the total carbohydrates, 92% is contributed by L-arabinose (Pramod and Venkatesh, 2006). *Lactobacillus* strains isolated from young people fermented more disaccharides that are used in the food industry and at home as sweeteners, such as corn syrup and honey.

Metabolomic characterization of the strains was performed in culture media, indicating that it may not accurately describe the *in situ* activity of intestinal microbes. Additionally, although hundreds of metabolites are targeted, the approach means that some metabolites that differ significantly between the groups may have been missed. Despite these shortcomings, the results clearly indicate differences in lipid metabolism.

Lipids dominate the list of the most significantly changed metabolites. It may be partially because lipids form the largest class of metabolites measured. However, a certain lipid subtype, acylcarnitines, was overrepresented in the list of significantly changed metabolites, indicating that their presence could not occur by chance alone. Moreover, contrary to other lipids, acylcarnitines cannot be explained by bacteria of different fermentation types or ecological origins. In the host, acylcarnitine esters are substrates and intermediates of fatty acid catabolism (Dambrova et al., 2022). The levels of many acylcarnitines were elevated in the presence of centenarian strains, suggesting an enhanced fatty acid catabolism.

The observed increase in arachidonic acid levels was unexpected, as arachidonic acid is an omega-6 polyunsaturated fatty acid that is often associated with proinflammatory processes and increased health risks. However, it should be noted that inflammatory signaling is primarily mediated by downstream metabolites such as prostaglandins and other oxidized derivatives, rather than by arachidonic acid itself. As our metabolomic analyses were performed under *in vitro* conditions, no conclusions could be drawn regarding the production of inflammatory mediators or their physiological relevance *in vivo*, and these aspects warrant further investigation.

Although the elevated ratio of arachidonic acid to other polyunsaturated fatty acids has received considerable attention in the context of chronic inflammation, arachidonic acid is also an essential fatty acid with important biological functions. Emerging evidence suggests that, in specific contexts, arachidonic acid may exert beneficial effects. For example, it is required for normal central nervous system development in infancy and is therefore included in infant formulas (Tallima and El Ridi, 2018; Crawford et al., 2023). Additionally, supplementation with arachidonic acid has been reported to improve age-related cognitive function in the elderly (Tokuda et al., 2014).

In the context of the present study, these findings are referenced only to illustrate the potential complexity of arachidonic acid biology. Although the lactobacilli analyzed here were isolated from centenarians with preserved cognitive function, any relationship between the bacterial metabolite production observed *in vitro*, polyunsaturated fatty acid balance, and healthy aging remains speculative and should be interpreted with caution.

Bile acids are one of the most recognized host-microbiota interactions. The primary bile acids (cholic acid and chenodeoxycholic acid) are metabolized in the intestinal lumen to secondary bile acids (deoxycholic acid and lithocholic acid) by the microbiota, and these modified bile acids are reabsorbed and reused by the host. Associations between bile acids and aging have been recognized; in particular, lithocholic acid has emerged as an anti-aging metabolite (Jin et al., 2024; Sinclair, 2025). Our results agree that lithocholic acid production might be beneficial for healthy aging, and this capacity depends on the strain origin and fermentation type.

A limitation of our study is the relatively small number of lactobacilli strains included in the biochemical and metabolomic analyses. This was related to one of the main aims of this study, namely, the identification of candidate strains suitable for probiotic development. According to the European Food Safety Authority (EFSA) guidelines, antibiotic resistance represents a key exclusion criterion for probiotic strains. More than three-quarters of the isolated strains were resistant to one or more antibiotics and were therefore excluded from further analyses. Consequently, only approximately one-quarter of the isolated strains of lactobacilli were subjected to detailed biochemical characterization and metabolomic profiling. The limited number of strains reduced the statistical power of the group comparisons, and the results should therefore be interpreted with caution. The analyses were exploratory in nature and may not fully reflect the results that might emerge in larger and more diverse strain collections. We hope that future studies conducted by independent research groups will confirm our findings using lactobacilli isolated from centenarians.

Conclusion: This is the first study comparing the microbiota of centenarians and young people, revealing different species compositions and metabolic profiles and allowing the identification of suitable probiotic candidates. In centenarians, there is an increased proportion of lactobacilli in their microbiota, and their microbiota is more diverse compared to that of young people. Antibiotic resistance of isolated lactobacilli depends on their habitat/ecological niche (vertebrate host-adapted, insect-adapted, nomadic, environmental, or free-living) and fermentation groups (OHEL, OHOL, FHEL). The carbohydrate fermentation and metabolome products of lactobacilli isolated from centenarians and young people are different. Lactobacilli isolated from centenarians have a “richer” metabolism, which is important for healthy aging and gives them an advantage for developing new probiotics.

## Data Availability

The data presented in this study are publicly available. The data can be found here: https://www.ncbi.nlm.nih.gov/genbank, accession numbers OL434975-OL435034 and OL435036-OL435052.
