# Supplementary material for: Culture-based studies of intestinal lactobacilli in young people and centenarians
Source: Front Microbiol. 2026 Mar 10;17:1746411. doi: 10.3389/fmicb.2026.1746411 (PMC13008948; doi:10.3389/fmicb.2026.1746411)
Supplement: Supplementary file 1 [file Table_1.pdf]

Supplementary table 1. Gut microbiota of centenarians and young people: prevalence (nr of subjects) and counts of different bacterial families (range, median in log_{10}; CFU/g) by cultural methods.

Phylum	Families	Centenarians		Young people	
		Prevalence<br>Nr of subjects	Counts min-max (median)	Prevalence<br>Nr of subjects	Counts min-max (median)
Actinobacteria	*Actinomycetaceae*	2	0-7.8 (0)	1	0-7.5 (0)
	Bifidobacteriaceae	13[1]	0-10.3 (4.5)	21[1]	0-10.3 (8)
	Coriobacteriaceae	19	0-9.5 (7.5)[2]	22	0-10.5 (8.6)[2]
	Eggerthellaceae	0	0	1	0-9 (0)
	Micrococcaceae	1	0-8 (0)	0	0
	Propionibacteriaceae	6	0-10 (0)	2	0-9 (0)
Bacteroidetes	*Bacteroidaceae*	22	0-10.4 (8)	24	0-11 (8.1)
	Odoribacteraceae	1	0-9.5 (0)	0	0
	Porphyromonadaceae	3	0-8.8 (0)	2	0-9 (0)
	Prevotellaceae	0	0	2	0-8.5 (0)
	Rikenellaceae	4	0-9.5 (0)	3	0-9 (0)
Firmicutes	*Christensenellaceae*	0	0	1	0-7.3 (0)
	Clostridiaceae	12	0-10.1 (0)	8	0-9 (0)
	Enterococcaceae	19	0-11 (5.9)[3]	13	0-7.8 (3.7)[3]
	Erysipelotrichaceae	2	0-7.6 (0)	7	0-10 (0)
	Eubacteriaceae	0	0	3	0-7.5 (0)
	Lachnospiraceae	3	0-8 (0)	0	0
	Lactobacillaceae	20	0-9 (6)	18	0-7.9 (5)
	Leuconostocaceae	2	0-6 (0)	2	0-6 (0)
	Peptoniphilaceae	1	0-8 (0)	1	0-8.5 (0)
	Peptostreptococcacea	1	0-6 (0)	1	0-6.8 (0)
	Ruminococcaceae	2	0-6.3 (0)	1	0-7.5 (0)
	Staphylococcaceae	5	0-10 (0)	2	0-9 (0)
	Streptococcaceae	14	0-9.6 (5)	15	0-9.5 (5)
	Veillonellaceae	1	0-8 (0)	1	0-8 (0)
Proteobacteria	*Burkholderiaceae*	1	0-10 (0)	0	0
	Enterobacteriaceae	22	0-9.1 (7)	21	0-8.3 (6)
	Hafniaceae	1	0-5.9 (0)	0	0
	Morganellaceae	2	0-8 (0)	1	0-5.3 (0)
	Neisseriaceae	0	0	1	0-8.5 (0)
	Pseudomonadaceae	1	0-7 (0)	0	0
	Zoogloeaceae	1	0-8.3 (0)	1	0-8.3 (0)

[1]p=0.032; [2]p=0.0031; [3]p=0.019
