# Supplementary material for: Culture-based studies of intestinal lactobacilli in young people and centenarians
Source: Front Microbiol. 2026 Mar 10;17:1746411. doi: 10.3389/fmicb.2026.1746411 (PMC13008948; doi:10.3389/fmicb.2026.1746411)
Supplement: Supplementary file 2 [file Table_2.pdf]

Supplementary table 2.

Origin of lactobacilli (isolation form old or young people; fermentation group: FHEL, OHEL, OHOL; origin: N-nomadic, E-environmental; V-veretebarete host)

and their minimum inhibitory concentrations (MIK) for various antibiotics

Species of lactobacillus	Old / young	fermentation group	orgin	Ampicillin (MIC)	Vancomycin (MIC)	Gentamycin (MIC)	Kanamycin (MIC)	Erytromycin (MIC)	Tertracyclin (MIC)	Clindamycin (MIC)	Chloramphenicol (MIC)	Strptomycin (MIC)
Lacticaseibacillus rhamnosus	old	FHEL	R	0.38	>256	2	96	0.125	0.75	1	2	12
Lacticaseibacillus paracasei	old	FHEL	R	0.38	>256	2	48	0.094	0.5	0.064	4	8
Lentilactobacillus parabuchneri	old	OHEL	K	1	>256	0.19	6	0.094	12	<0,016	<0,016	2
Lacticaseibacillus zeae	old	FHEL	R	0.5	>256	2	64	0.125	0.75	1	2	12
Ligilactobacillus salivarius	old	OHOL	S	0.25	>256	4	>256	0.25	1	0.016	1.5	32
Limosilactobacillus reuteri	old	OHEL	S	0.5	>256	1.5	32	0.75	>256	0.125	6	8
Lacticaseibacillus rhamnosus	old	FHEL	R	0.25	>256	2	96	0.094	0.38	0.25	2	12
Lacticaseibacillus casei	old	FHEL	R	0.19	>256	2	64	0.064	0.75	0.064	2	12
Lacticaseibacillus rhamnosus	old	FHEL	R	0.75	>256	2	32	0.25	0.5	0.38	3	8
Lacticaseibacillus rhamnosus	old	FHEL	R	0.75	>256	2	32	0.19	0.5	0.38	3	8
Lactobacillus gasseri	old	OHOL	S	0.125	0.75	4	64	0.094	0.75	2	4	4
Limosilalactobacillus fermentum	old	OHEL	SO	0.094	>256	2	96	0.5	2	0.032	3	16
Lacticaseibacillus rhamnosus	old	FHEL	R	0.38	>256	3	96	0.125	0.5	0.19	4	12
Lacticaseibacillus rhamnosus	old	FHEL	R	0.5	>256	3	32	0.125	0.75	0.38	4	8

Lactobacillus gasseri	old	OHOL	S	0.064	0.75	0.75	6	<0,016	0.5	0.25	1.5	0.38
Limosilactobacillus frumenti	old	OHEL	S	0.064	1.5	0.75	12	0.064	3	0.016	2	6
Lacticaseibacillus rhamnosus	old	FHEL	R	>256	>256	3	48	0.25	0.75	0.38	3	8
Lactobacillus gasseri	old	OHOL	S	0.047	0.5	1	12	<0,016	0.5	0.5	1.5	0.75
Limosilalactobacillus fermentum	old	OHEL	SO	0.094	192	2	48	0.38	3	0.032	3	16
Latilactobacillus sakei	old	FHEL	K	0.5	24	1	2	0.094	1	<0.016	1.5	6
Lacticaseibacillus paracasei	old	FHEL	R	0.38	>256	3	128	0.19	0.75	0.094	3	16
Limosilactobacillus frumenti	old	OHEL	S	0.38	1	0.5	8	0.064	3	0.016	2	6
Ligilactobacillus salivarius	old	OHOL	S	0.25	>256	4	>256	0.5	1	0.125	2	48
Limosilactobacillus mucosae	old	OHEL	S	0.125	>256	0.5	16	0.19	6	0.016	2	4
Ligilactobacillus ruminis	old	OHOL	S	0.5	>256	4	128	0.125	1	0.094	1.5	12
Lactobacillus gasseri	old	OHOL	S	0.125	0.75	2	48	0.047	0.75	2	1.5	2
Lactobacillus gasseri	old	OHOL	S	0.125	0.75	2	48	0.094	0.75	3	3	2
Limosilactobacillus mucosae	old	OHEL	S	0.125	>256	12	96	1.5	12	6	2	128
Lacticaseibacillus paracasei	old	FHEL	R	0.75	>256	2	32	0.19	0.75	0.094	4	16
Lacticaseibacillus paracasei	old	FHEL	R	0.5	>256	3	96	0.094	0.5	0.064	3	16
Lacticaseibacillus rhamnosus	old	FHEL	R	0.38	>256	3	32	0.094	0.5	0.19	3	12
Schleiferilactobacillus harbensis	old	FHEL	K	0.25	32	1	16	0.094	0.25	0.5	3	1.5
Ligilactobacillus ruminis	old	OHOL	S	0.75	>256	4	>256	0.25	1	0.064	2	24
Lactobacillus gasseri	old	OHOL	S	0.125	1	2	128	0.094	1	2	2	6
Lacticaseibacillus paracasei	old	FHEL	R	0.25	>256	2	64	0.125	0.5	0.032	2	12
Ligilactobacillus salivarius	old	OHOL	S	0.25	>256	4	>256	0.5	1.5	0.047	1	48
Ligilactobacillus salivarius	old	OHOL	S	0.38	>256	3	>256	0.75	1.5	0.047	2	64
Lactobacillus gasseri	old	OHOL	S	0.19	1	3	>256	0.094	1.5	1.5	4	4
Lacticaseibacillus paracasei	old	FHEL	R	0.38	>256	1.5	64	0.094	0.75	0.047	2	12
Ligilactobacillus ruminis	old	OHOL	S	0.38	>256	4	>256	0.125	0.75	0.19	2	32
Ligilactobacillus salivarius	old	OHOL	S	0.19	>256	64	>256	0.19	0.5	0.032	1.5	256
Lactobacillus acidophilus	old	OHOL	S	0.19	0.38	2	32	0.094	0.38	0.25	3	2

Lacticaseibacillus rhamnosus	old	FHEL	R	0.25	>256	2	32	0.125	0.5	0.38	4	4
Ligilactobacillus salivarius	old	OHOL	S	0.25	>256	3	192	0.5	1.5	0.032	1.5	24
Lacticaseibacillus rhamnosus	old	FHEL	R	0.5	>256	2	32	0.38	0.75	0.25	2	8
Lacticaseibacillus paracasei	old	FHEL	R	0.25	>256	1	32	0.064	0.25	0.047	1	3
Limosilalactobacillus fermentum	young	OHEL	SO	0.19	>256	2	32	1	3	0.047	3	16
Lactobacillus acidophilus	young	OHOL	S	0.125	0.5	1	48	0.047	0.5	0.5	4	2
Lacticaseibacillus rhamnosus	young	FHEL	R	0.38	>256	2	64	0.19	0.5	0.38	4	6
Lacticaseibacillus paracasei	young	FHEL	R	0.5	>256	4	>256	0.064	0.75	0.125	4	24
Lactobacillus acidophilus	young	OHOL	S	0.19	0.38	1.5	64	0.125	0.5	0.5	4	2
Ligilactobacillus ruminis	young	OHOL	S	0.38	>256	4	>256	0.19	1	0.125	2	12
Lacticaseibacillus paracasei	young	FHEL	R	0.5	>256	2	64	0.125	0.75	0.047	4	8
Lacticaseibacillus rhamnosus	young	FHEL	R	0.75	>256	3	64	0.125	0.5	0.25	4	12
Lacticaseibacillus rhamnosus	young	FHEL	R	0.38	>256	2	48	0.19	0.75	0.38	4	4
Lactobacillus gasseri	young	OHOL	S	0.064	1	0.75	32	0.047	0.5	0.25	1.5	2
Lentilactobacillus otakiensis	old	OHEL	K	0.38	>256	0.125	2	0.064	3	<0,016	0.023	1.5
Ligilactobacillus ruminis	old	OHOL	S	0.75	>256	6	>256	0.125	0.75	0.125	1.5	24
Limosilactobacillus mucosae	old	OHEL	S	0.047	>256	1	32	0.38	16	0.032	3	8
Lactobacillus gasseri	young	OHOL	S	0.19	0.75	2	64	0.25	1	3	2	3
Lacticaseibacillus paracasei	young	FHEL	R	0.5	>256	3	>256	0.125	0.75	0.25	3	24
Lacticaseibacillus paracasei	young	FHEL	R	0.75	>256	2	48	0.064	0.5	0.032	3	12
Lacticaseibacillus rhamnosus	young	FHEL	R	0.38	>256	2	32	0.125	0.38	0.38	2	6
Lactobacillus gasseri	old	OHOL	S	0.016	0.38	0.5	6	0.023	0.19	0.75	1.5	0.5
Ligilactobacillus ruminis	young	OHOL	S	1	>256	6	>256	0.25	0.5	0.125	2	32
Ligilactobacillus ruminis	young	OHOL	S	0.38	>256	8	>256	0.125	1	0.064	2	32
Lacticaseibacillus rhamnosus	young	FHEL	R	0.25	>256	2	32	0.064	0.38	0.38	3	3
Lactobacillus gasseri	young	OHOL	S	0.19	1	2	48	0.032	0.75	2	3	2
Ligilactobacillus ruminis	young	OHOL	S	1	>256	1	12	0.094	0.5	0.032	3	8
Laticasebacillus paracasei	young	FHEL	R	0.38	>256	1	12	0.064	0.5	0.032	2	4

Lactobacillus gasseri	young	OHOL	S	0.047	0.5	1.5	24	0.047	0.19	0.094	2	1
Ligilactobacillus ruminis	young	OHOL	S	0.25	>256	2	96	0.125	0.75	0.094	1.5	12
Lactobacillus acidophilus	young	OHOL	S	0.19	0.25	2	32	0.047	0.5	0.25	2	2
Lacticaseibacillus rhamnosus	young	FHEL	R	0.38	>256	2	32	0.19	0.5	0.5	4	4
Lactobacillus acidophilus	young	OHOL	S	0.19	0.38	2	32	0.047	0.38	0.25	3	2
Latilactobacillus curvatus	young	FHEL	K	0.125	>256	2	8	0.125	0.75	<0.016	3	32
Lactobacillus delbrueckii	young	OHOL	S	0.064	0.38	1	48	0.032	0.5	0.032	2	3
