# Supplementary material for: Culture-based studies of intestinal lactobacilli in young people and centenarians
Source: Front Microbiol. 2026 Mar 10;17:1746411. doi: 10.3389/fmicb.2026.1746411 (PMC13008948; doi:10.3389/fmicb.2026.1746411)
Supplement: Supplementary file 4 [file Table_4.pdf]

Supplementary table 4

Measured metabolites and change in their relative abundance
(ratio to empty growth media) in growth media depending on lactobacilli strains.

		Centenarians	Younger controls	Effect size (Cohen's d)	p-value	association with bacterial origin	association with fermentative type
**Abbreviation or name**	**name**						
**Acylcarnitines**							
C0	Carnitine	0.96±0.2	0.80±0.1	1.07	0.022066	-	-
C2	Acetylcarnitine	1.19±0.2	1.05±0.2	0.58	0.315399	-	-
C3	Propionylcarnitine	1.07±0.3	0.69±0.2	1.36	0.020611	-	-
C3-DC (C4-OH)	Sum of Malonylcarnitine and Hydroxybutyrylcarnitine	0.61±0.2	0.51±0.1	0.54	0.264993	*	**
C3-OH	Hydroxypropionylcarnitine	1.45±0.2	1.28±0.2	0.76	0.181013	-	-
C3:1	Propenoylcarnitine	1.25±0.3	1.18±0.2	0.29	0.546526	-	-
C4	Butyrylcarnitine	1.18±0.3	0.92±0.1	1.11	0.01856	-	-
C4:1	Butenylcarnitine	0.84±0.3	0.64±0.2	0.80	0.148131	-	-
C5	Valerylcarnitine	2.46±1.6	1.20±1.1	0.88	0.100895	-	-
C5-DC (C6-OH)	Sum of Glutarylcarnitine and Hydroxyhexanoylcarnitine	1.66±0.4	1.47±0.2	0.60	0.18788	-	-
C5-M-DC	Methylglutarylcarnitine	0.77±0.2	0.63±0.2	0.67	0.218821	*	-
C5-OH (C3-DC-M)	Sum of Hydroxyvalerylcarnitine and Methylmalonylcarnitine	1.12±0.2	1.00±0.1	0.84	0.063146	-	-
C5:1	Tiglylcarnitine	0.85±0.3	0.46±0.2	1.37	0.014849	-	-
C5:1-DC	Glutaconylcarnitine	1.38±0.4	1.23±0.2	0.49	0.328336	-	-
C6 (C4:1-DC)	Sum of Hexanoylcarnitine and Fumarylcarnitine	1.00±0.3	0.77±0.2	1.01	0.059227	-	-

C6:1	Hexenoylcarnitine	0.84±0.2	0.68±0.1	0.88	0.085158	-	-
C7-DC	Pimeloylcarnitine	1.25±0.5	1.20±0.5	0.08	0.88819	-	-
C9	Nonaylcarnitine	0.84±0.2	0.62±0.1	1.38	0.017513	-	-
C10	Decanoylcarnitine	0.64±0.2	0.56±0.1	0.43	0.361935	***	***
C10:1	Decenoylcarnitine	0.98±0.2	1.12±0.2	0.66	0.254828	-	-
C10:2	Decadienoylcarnitine	0.97±0.1	0.77±0.1	1.65	0.012832	-	-
C12	Dodecanoylcarnitine	1.44±0.4	1.16±0.2	0.91	0.056461	-	-
C12-DC	Dodecanedioylcarnitine	1.14±0.1	1.11±0.0	0.28	0.516612	*	*
C12:1	Dodecenoylcarnitine	0.93±0.2	1.07±0.2	0.64	0.284235	-	-
C14	Tetradecanoylcarnitine	0.91±0.5	0.66±0.1	0.70	0.106639	-	-
C14:1	Tetradecenoylcarnitine	0.81±0.2	0.82±0.1	0.05	0.920339	-	-
C14:1-OH	Hydroxytetradecenoylcarnitine	1.16±0.8	1.03±0.3	0.19	0.675966	*	-
C14:2	Tetradecadienoylcarnitine	1.20±0.1	1.16±0.1	0.32	0.539897	-	-
C14:2-OH	Hydroxytetradecadienoyl- carnitine	0.53±0.2	0.55±0.1	0.13	0.800801	-	-
C16	Hexadecanoylcarnitine	0.18±0.1	0.09±0.0	0.99	0.03455	***	**
C16-OH	Hydroxyhexadecanoylcarnitine	0.68±0.2	0.62±0.1	0.39	0.433659	-	-
C16:1	Hexadecenoylcarnitine	0.78±0.1	0.75±0.2	0.21	0.708454	-	-
C16:1-OH	Hydroxyhexadecenoylcarnitine	1.18±0.4	1.14±0.4	0.09	0.86195	-	-
C16:2	Hexadecadienoylcarnitine	0.44±0.2	0.31±0.0	0.76	0.079251	-	-
C16:2-OH	Hydroxyhexadecadienoyl- carnitine	0.83±0.2	0.80±0.1	0.29	0.524958	**	*
C18	Octadecanoylcarnitine	0.91±0.5	0.66±0.1	0.66	0.129358	-	-
C18:1	Octadecenoylcarnitine	0.88±0.3	0.82±0.1	0.32	0.474274	*	*
C18:1-OH	Hydroxyoctadecenoylcarnitine	1.07±0.3	1.09±0.2	0.06	0.9048	-	-
C18:2	Octadecadienylcarnitine	0.93±0.3	0.61±0.2	1.15	0.025211	-	-
**Amino acids**							
Ala	Alanine	0.39±0.3	0.27±0.1	0.52	0.238133	***	***
Arg	Arginine	0.23±0.1	0.27±0.0	0.39	0.376666	***	***
Asn	Asparagine	0.31±0.3	0.17±0.1	0.54	0.219232	***	***
Asp	Aspartate	0.60±0.2	0.52±0.1	0.42	0.337572	**	**
Cys	Cysteine	1.24±0.4	1.22±0.5	0.04	0.942687	*	-
Glu	Glutamate	0.61±0.3	0.49±0.1	0.53	0.212833	***	***
Gly	Glycine	0.48±0.3	0.36±0.1	0.55	0.221866	***	***

His	Histidine	0.50±0.3	0.35±0.1	0.55	0.202457	***	***
Ile	Isoleucine	0.51±0.2	0.40±0.1	0.53	0.295118	***	***
Leu	Leucine	0.45±0.2	0.37±0.1	0.45	0.370595	***	***
Lys	Lysine	0.20±0.3	0.11±0.1	0.41	0.355356	***	***
Met	Methionine	0.39±0.4	0.25±0.1	0.47	0.26785	***	***
Phe	Phenylalanine	0.48±0.3	0.36±0.1	0.53	0.23644	***	***
Pro	Proline	0.85±0.2	0.71±0.1	0.81	0.094306	*	*
Ser	Serine	0.37±0.3	0.24±0.0	0.62	0.147426	***	***
Thr	Threonine	0.42±0.3	0.29±0.1	0.49	0.265841	***	***
Trp	Tryptophan	0.45±0.3	0.32±0.1	0.55	0.207569	***	***
Tyr	Tyrosine	0.47±0.3	0.29±0.1	0.67	0.124981	***	***
Val	Valine	0.46±0.3	0.32±0.1	0.55	0.209837	***	***
**Amino acid derivatives**							
1-Met-His	1-Methylhistidine	0.66±0.3	0.40±0.1	1.01	0.030202	***	**
3-Met-His	3-Methylhistidine	0.62±0.3	0.49±0.1	0.51	0.252969	**	***
5-AVA	5-Aminovaleric acid	0.46±0.3	0.34±0.1	0.49	0.256307	***	***
AABA	α-Aminobutyric acid	0.45±0.3	0.32±0.1	0.62	0.160213	***	***
ADMA	Asymmetric dimethylarginine	0.43±0.4	0.23±0.1	0.65	0.138234	***	***
alpha-AAA	α-Aminoadipic acid	0.61±0.4	0.40±0.2	0.56	0.249921	-	-
Anserine	Anserine	0.67±0.4	0.62±0.2	0.13	0.771013	***	***
Betaine	Betaine	1.02±0.1	0.94±0.1	0.63	0.199033	-	-
c4-OH-Pro	cis-4-Hydroxyproline	0.92±0.3	0.76±0.2	0.63	0.197148	-	-
Cit	Citrulline	0.21±0.1	1.06±1.6	0.65	0.35952	*	-
Creatinine	Creatinine	1.18±0.1	1.14±0.1	0.41	0.435777	**	-
Cystine	Cystine	0.76±0.3	1.87±1.5	0.91	0.218022	-	-
HArg	Homoarginine	1.62±0.7	1.18±0.2	0.76	0.09108	-	-
HCys	Homocysteine	7.18±7.7	11.77±7.4	0.56	0.321879	***	**
Kynurenine	Kynurenine	0.54±0.3	0.43±0.1	0.50	0.242577	**	**
Orn	Ornithine	0.98±2.1	0.12±0.1	0.57	0.166535	***	***
PAG	Phenylacetylglycine	1.02±0.9	0.77±0.7	0.29	0.374175	-	-
PheAlaBetaine	Phenylalanine betaine	0.60±0.5	0.71±0.2	0.68	0.172806	***	***
ProBetaine	Proline betaine	0.76±0.1	0.64±0.0	1.12	0.016895	*	**

Sarcosine	Sarcosine	0.57±0.3	0.51±0.2	0.23	0.654564	**	*
SDMA	Symmetric dimethylarginine	0.54±0.3	0.39±0.1	0.71	0.104235	***	***
t4-OH-Pro	trans-4-Hydroxyproline	2.06±0.9	1.49±0.6	0.72	0.173143	-	-
Taurine	Taurine	0.47±0.3	0.35±0.1	0.52	0.235255	***	***
TrpBetaine	Tryptophan betaine	1.60±0.5	1.41±0.4	0.41	0.450341	-	-
beta-Ala	β-Alanine	0.40±0.3	0.29±0.1	0.49	0.260409	***	***
GABA	γ-Aminobutyric acid	0.41±0.3	0.27±0.1	0.57	0.186211	***	***
Histamine	Histamine	0.50±0.3	0.34±0.1	0.65	0.167422	***	***
Putrescine	Putrescine	0.25±0.3	0.12±0.1	0.45	0.307667	***	***
Serotonin	Serotonin	0.92±0.3	0.78±0.2	0.59	0.236878	-	-
Spermidine	Spermidine	0.17±0.3	0.07±0.1	0.46	0.291453	***	***
Spermine	Spermine	0.62±0.3	0.82±0.2	0.68	0.178939	-	-
**Bile acids**							
CA	Cholic acid	1.24±0.3	0.95±0.2	1.04	0.048746	**	-
CDCA	Chenodeoxycholic acid	0.84±0.5	0.95±0.5	0.20	0.730611	-	*
DCA	Deoxycholic acid	1.23±0.6	0.76±0.6	0.73	0.222727	-	-
GCA	Glycocholic acid	1.02±0.7	0.86±0.3	0.28	0.548997	-	-
GLCA	Glycolithocholic acid	1.77±1.4	0.80±0.4	0.89	0.049416	***	***
GLCAS	Glycolithocholic acid sulfate	0.31±0.3	0.33±0.2	0.09	0.8601	***	***
TCA	Taurocholic acid	0.71±0.4	0.72±0.2	0.05	0.916759	-	-
TCDCA	Taurochenodeoxycholic acid	0.58±0.7	0.83±0.7	0.33	0.565001	-	-
TMCA	Tauromurocholic acid	0.73±0.4	0.72±0.2	0.02	0.998	-	-
**Ceramides, hexylceramides and dihexylceramides with indicated fatty acid residues**							
Cer(d16:1/18:0)		0.48±0.3	0.52±0.3	0.10	0.866358	-	-
Cer(d16:1/20:0)		0.56±0.5	0.76±0.5	0.37	0.512454	-	-
Cer(d16:1/22:0)		0.75±0.6	0.39±0.3	0.73	0.137054	-	*
Cer(d16:1/23:0)		0.86±0.6	0.93±0.5	0.11	0.824214	-	-
Cer(d18:1/14:0)		1.33±0.3	0.94±0.5	0.89	0.166708	-	-
Cer(d18:1/18:0(OH))		0.81±0.5	0.89±0.4	0.16	0.760784	-	-
Cer(d18:1/18:1)		0.76±0.4	0.57±0.4	0.46	0.438952	-	-
Cer(d18:1/20:0(OH))		0.59±0.4	0.69±0.4	0.24	0.678689	-	-
Cer(d18:1/23:0)		0.89±1.0	0.67±0.6	0.25	0.604507	-	-

Cer(d18:1/24:1)		0.73±1.1	1.21±1.5	0.34	0.568355	-	-
Cer(d18:2/14:0)		1.04±0.4	0.91±0.5	0.28	0.633456	-	-
Cer(d18:2/16:0)		0.51±0.2	0.54±0.2	0.16	0.77774	-	-
Cer(d18:2/18:0)		0.62±0.6	0.57±0.3	0.10	0.833273	*	-
Cer(d18:2/18:1)		1.06±0.5	0.85±0.1	0.53	0.223512	-	-
Cer(d18:2/20:0)		0.68±0.3	0.51±0.5	0.38	0.537733	-	-
Cer(d18:2/22:0)		1.33±0.7	1.40±0.8	0.08	0.892848	-	-
Cer(d18:2/23:0)		0.77±0.4	0.64±0.6	0.21	0.719603	-	-
Cer(d18:2/24:0)		0.72±0.5	2.02±1.4	1.13	0.130081	-	-
Cer(d18:2/24:1)		0.78±0.3	0.83±0.5	0.11	0.855617	-	-
Cer(d18:0/18:0(OH))		0.72±0.5	0.66±0.4	0.14	0.7919	-	-
Cer(d18:0/18:0)		0.92±0.2	0.88±0.3	0.14	0.80457	-	-
Cer(d18:0/20:0)		0.74±0.5	0.80±0.3	0.13	0.78961	-	-
Cer(d18:0/22:0)		0.72±0.4	0.46±0.2	0.75	0.125612	-	-
Cer(d18:0/24:0)		0.84±0.4	0.52±0.4	0.70	0.223114	*	*
Cer(d18:0/24:1)		1.32±0.5	1.33±0.3	0.02	0.962931	-	-
Cer(d18:0/26:1(OH))		0.93±0.6	1.04±0.7	0.14	0.796775	-	-
Cer(d18:0/26:1)		1.78±1.3	1.23±1.4	0.37	0.522997	-	-
Hex2Cer(d18:1/14:0)		0.63±0.3	0.68±0.7	0.09	0.895181	*	-
Hex2Cer(d18:1/16:0)		0.74±1.0	0.86±1.1	0.10	0.856406	**	-
Hex2Cer(d18:1/18:0)		0.38±0.2	0.34±0.4	0.11	0.857564	-	-
Hex2Cer(d18:1/20:0)		0.63±0.5	0.20±0.2	1.15	0.015816	-	-
Hex2Cer(d18:1/22:0)		1.58±2.2	3.97±3.4	0.76	0.247486	-	-
Hex2Cer(d18:1/24:0)		1.26±1.3	1.79±1.6	0.33	0.573646	**	*
Hex2Cer(d18:1/26:0)		9.05±6.0	13.98±5.2	0.82	0.152229	*	-
Hex3Cer(d18:1/16:0)		0.89±0.8	2.16±1.3	1.08	0.122189	-	-
Hex3Cer(d18:1/20:0)		0.53±0.5	0.40±0.4	0.29	0.577257	-	-
Hex3Cer(d18:1/22:0)		0.77±0.8	0.49±0.5	0.40	0.424658	-	-
Hex3Cer(d18:1/24:1)		0.50±0.4	0.79±1.0	0.35	0.591132	*	-
Hex3Cer(d18:1/26:1)		0.41±0.3	0.90±0.5	1.01	0.1426	-	-
HexCer(d16:1/20:0)		2.30±2.3	3.53±1.2	0.64	0.188163	-	-
HexCer(d16:1/22:0)		0.53±0.5	0.97±0.7	0.65	0.307368	-	-

HexCer(d16:1/24:0)		0.71±0.6	0.52±0.5	0.33	0.5333	-	-
HexCer(d18:1/14:0)		1.03±0.8	0.44±0.3	0.98	0.036325	-	-
HexCer(d18:1/16:0)		0.92±0.5	0.53±0.3	0.84	0.114883	*	*
HexCer(d18:1/18:1)		3.73±1.1	2.96±1.6	0.50	0.421932	-	-
HexCer(d18:1/20:0)		0.55±0.6	0.93±0.6	0.56	0.325195	-	-
HexCer(d18:1/22:0)		0.84±0.6	0.96±0.4	0.22	0.668084	*	-
HexCer(d18:1/23:0)		0.58±0.4	0.59±0.4	0.03	0.951795	-	-
HexCer(d18:1/24:0)		1.23±1.1	0.98±0.7	0.26	0.60127	-	**
HexCer(d18:1/24:1)		1.96±0.9	1.26±0.8	0.77	0.182568	-	-
HexCer(d18:1/26:0)		2.71±2.2	3.10±3.9	0.11	0.858042	-	-
HexCer(d18:1/26:1)		0.77±0.9	0.83±0.7	0.07	0.894811	-	-
HexCer(d18:2/16:0)		0.88±0.3	0.63±0.3	0.74	0.2193	***	*
HexCer(d18:2/18:0)		0.85±0.8	0.75±0.4	0.15	0.745927	-	-
HexCer(d18:2/20:0)		1.43±1.1	1.16±0.7	0.28	0.582923	-	-
HexCer(d18:2/22:0)		0.72±0.5	0.76±0.4	0.08	0.878264	-	-
HexCer(d18:2/23:0)		1.34±1.1	1.22±0.8	0.12	0.821009	-	-
HexCer(d18:2/24:0)		0.83±0.7	0.86±0.5	0.05	0.920213	-	-
**Cholesterol esters with indicated fatty acid residues**							
CE(14:1)		0.81±1.0	1.28±1.0	0.46	0.421312	*	*
CE(15:1)		0.90±0.4	0.80±0.3	0.25	0.636277	-	-
CE(17:1)		0.18±0.2	0.64±0.2	2.01	0.013448	-	-
CE(18:0)		1.06±0.4	0.76±0.5	0.61	0.312746	-	-
CE(18:3)		0.41±0.5	0.56±0.3	0.36	0.465577	-	-
CE(20:0)		7.86±5.7	4.65±6.2	0.49	0.397935	-	-
CE(20:1)		1.71±1.6	0.91±0.8	0.61	0.199548	-	-
CE(20:3)		0.56±0.4	0.34±0.4	0.52	0.380076	-	-
CE(20:5)		1.00±0.0	1.00±0.0	0.00	1	-	-
CE(22:0)		0.88±0.6	0.81±0.5	0.13	0.809529	*	-
CE(22:1)		0.47±0.4	0.43±0.3	0.12	0.820518	-	-
CE(22:2)		0.62±0.4	0.28±0.3	0.84	0.120265	-	-
CE(22:5)		0.97±0.4	0.48±0.3	1.21	0.038063	-	-
CE(22:6)		0.85±0.4	0.66±0.5	0.36	0.537784	-	-

Diacylglycerides with indicated fatty acid residues							
DG(14:0_18:2)		1.09±0.2	1.02±0.2	0.31	0.541712	-	-
DG(14:0_20:0)		1.01±0.5	0.86±0.2	0.35	0.449332	*	-
DG(14:1_18:1)		0.96±0.6	0.55±0.7	0.56	0.33226	-	-
DG(14:1_20:2)		1.24±0.7	1.23±0.3	0.02	0.96329	-	-
DG(16:0_16:0)		1.87±0.5	2.55±1.2	0.66	0.328582	-	-
DG(16:0_18:2)		0.45±0.3	0.56±0.4	0.26	0.664651	-	-
DG(16:0_20:3)		2.50±1.6	1.90±1.1	0.40	0.42129	-	-
DG(16:0_20:4)		0.36±0.2	0.37±0.3	0.03	0.958754	*	-
DG(16:1_18:1)		1.07±1.2	1.01±0.3	0.06	0.888774	-	-
DG(16:1_18:2)		1.39±0.5	1.57±0.7	0.26	0.660863	-	-
DG(16:1_20:0)		0.32±0.2	0.59±0.3	0.99	0.108594	-	*
DG(17:0_17:1)		0.86±1.0	0.54±0.3	0.39	0.366718	-	-
DG(18:0_20:0)		1.34±0.5	1.49±0.2	0.40	0.360396	-	-
DG(18:0_20:4)		1.20±0.9	1.42±0.8	0.24	0.662113	-	-
DG(18:1_18:2)		0.83±0.3	0.69±0.6	0.28	0.659863	-	-
DG(18:1_18:3)		8.95±8.7	11.21±6.9	0.27	0.610615	-	-
DG(18:1_18:4)		0.61±0.3	0.47±0.3	0.41	0.445824	*	*
DG(18:1_20:0)		1.68±1.2	0.86±1.0	0.70	0.21005	-	-
DG(18:1_20:1)		1.03±0.4	1.58±0.6	0.98	0.145747	-	-
DG(18:1_20:2)		0.67±0.3	0.50±0.6	0.30	0.632412	**	-
DG(18:1_20:3)		0.82±0.5	1.10±0.6	0.47	0.421536	-	-
DG(18:1_20:4)		0.58±0.3	0.63±0.3	0.17	0.768271	-	-
DG(18:1_22:5)		1.32±0.5	1.17±0.4	0.29	0.602866	-	-
DG(18:1_22:6)		0.89±0.7	0.99±0.5	0.15	0.75884	-	-
DG(18:2_18:3)		3.50±2.9	2.94±1.5	0.23	0.625409	-	-
DG(18:2_18:4)		1.87±1.2	1.83±1.2	0.03	0.961017	-	-
DG(18:2_20:0)		0.85±0.2	0.74±0.3	0.35	0.574037	-	-
DG(18:2_20:4)		1.05±0.6	0.84±0.5	0.37	0.499992	-	-
DG(18:3_18:3)		0.93±0.3	0.76±0.4	0.45	0.428084	-	-
DG(18:3_20:2)		1.01±0.7	1.09±0.4	0.13	0.776861	-	-
DG(21:0_22:6)		1.69±0.9	1.78±1.4	0.07	0.904813	*	*

DG(22:1_22:2)		0.59±0.3	0.64±0.3	0.14	0.808256	-	-
**Lysophosphocholines with indicated fatty acid residues**							
lysoPC a C14:0		1.09±0.2	1.14±0.1	0.32	0.495221	-	-
lysoPC a C16:0		1.19±1.6	1.85±2.3	0.30	0.621378	-	-
lysoPC a C16:1		0.91±0.3	0.94±0.2	0.12	0.800788	**	-
lysoPC a C17:0		0.83±0.4	0.83±0.2	0.01	0.982109	-	-
lysoPC a C18:0		1.04±0.3	1.26±0.3	0.75	0.185689	*	-
lysoPC a C18:1		5.74±17.3	1.39±1.1	0.34	0.402288	-	-
lysoPC a C18:2		6.26±19.8	3.39±5.0	0.19	0.652105	-	-
lysoPC a C20:3		1.03±0.3	0.95±0.3	0.28	0.61704	-	*
lysoPC a C20:4		1.06±1.4	0.64±0.2	0.42	0.313618	-	-
lysoPC a C24:0		0.97±0.4	1.04±0.3	0.20	0.701986	-	-
lysoPC a C26:0		0.67±0.5	0.79±0.2	0.31	0.497944	-	-
lysoPC a C26:1		0.67±0.4	0.80±0.2	0.42	0.376621	-	-
lysoPC a C28:0		1.06±0.3	1.20±0.3	0.49	0.387188	-	-
lysoPC a C28:1		1.19±0.5	1.10±0.4	0.19	0.729277	-	-
**Phosphocholines with indicated summary fatty acid residues**							
PC aa C24:0		1.32±0.7	1.25±0.6	0.10	0.853428	-	-
PC aa C26:0		1.25±0.2	1.32±0.3	0.22	0.707744	-	-
PC aa C28:1		0.90±0.4	0.97±0.2	0.18	0.705234	-	-
PC aa C30:0		1.30±0.3	1.36±0.5	0.13	0.827711	-	-
PC aa C32:2		4.78±3.6	7.01±2.8	0.64	0.237459	-	-
PC aa C32:3		1.63±1.1	2.37±1.6	0.50	0.418781	-	*
PC aa C34:4		0.71±0.4	0.45±0.5	0.54	0.359814	-	-
PC aa C36:0		0.91±0.4	1.03±0.5	0.25	0.659779	-	-
PC aa C36:6		0.77±0.6	0.74±0.6	0.04	0.934911	-	-
PC aa C38:0		8.92±6.4	13.20±9.8	0.47	0.448708	-	-
PC aa C40:1		1.11±0.3	1.26±0.2	0.58	0.288132	-	-
PC aa C40:2		0.76±0.6	0.82±0.6	0.09	0.875994	-	-
PC aa C40:3		1.00±0.0	1.00±0.0	0.00	1	-	-
PC aa C42:0		1.07±0.4	1.19±0.4	0.24	0.665901	-	-
PC aa C42:1		0.35±0.2	0.48±0.1	0.72	0.166298	***	***

PC aa C42:2		1.21±0.3	1.31±0.3	0.29	0.605499	-	-
PC aa C42:4		0.76±0.5	0.94±0.5	0.33	0.553166	-	-
PC aa C42:6		1.32±0.3	1.09±0.3	0.63	0.283854	-	*
**Plasmalogens with indicated summary fatty acid residues**							
PC ae C30:0		1.21±0.3	1.44±0.4	0.64	0.302921	-	-
PC ae C30:1		0.45±0.3	0.31±0.3	0.44	0.457335	*	*
PC ae C30:2		1.30±0.7	1.20±0.3	0.20	0.658685	-	-
PC ae C32:2		1.05±0.4	0.88±0.4	0.40	0.45332	*	-
PC ae C34:0		0.84±0.9	0.67±0.5	0.22	0.647245	-	-
PC ae C34:2		1.65±1.5	2.06±1.6	0.25	0.658164	-	-
PC ae C34:3		1.72±1.8	0.32±0.6	0.99	0.03313	-	**
PC ae C36:0		1.61±0.7	1.56±0.5	0.08	0.875268	-	-
PC ae C38:0		1.21±0.2	1.35±0.2	0.54	0.348867	-	-
PC ae C38:3		1.00±0.0	1.00±0.0	0.00	1	-	-
PC ae C38:4		1.00±0.0	1.00±0.0	0.00	1	-	-
PC ae C40:1		0.69±0.4	0.86±0.4	0.40	0.493522	*	-
PC ae C40:3		0.25±0.3	0.42±0.4	0.45	0.470115	-	-
PC ae C40:4		1.00±0.4	1.12±0.5	0.27	0.649755	-	-
PC ae C40:6		1.04±0.4	1.31±0.3	0.71	0.170819	-	-
PC ae C42:0		1.33±0.3	1.51±0.3	0.50	0.390756	-	-
PC ae C42:1		0.94±0.3	0.94±0.3	0.02	0.974512	-	-
PC ae C42:2		1.47±0.8	1.99±0.5	0.76	0.138374	-	-
PC ae C42:3		2.79±1.6	2.79±0.7	0.00	0.999499	*	-
PC ae C42:5		1.26±0.3	1.44±0.3	0.63	0.289451	-	-
PC ae C44:3		1.46±0.5	1.76±0.5	0.56	0.318543	-	-
PC ae C44:4		1.54±0.2	1.63±0.5	0.20	0.750106	-	-
PC ae C44:5		0.82±0.2	0.97±0.3	0.55	0.349873	*	*
PC ae C44:6		1.21±0.2	1.44±0.4	0.72	0.265078	-	-
**Spingomyelins and hydroxysphingomyelins with indicated fatty acid residue**							
SM (OH) C14:1		4.19±4.4	4.23±3.2	0.01	0.987484	-	-
SM (OH) C24:1		0.66±0.8	0.25±0.2	0.66	0.134353	-	-
SM C16:1		1.12±0.9	2.73±1.6	1.11	0.118391	-	-

SM C20:2		0.94±1.0	0.99±0.5	0.07	0.886	-	-
SM C26:0		1.65±1.6	2.07±2.8	0.17	0.785907	**	-
SM C26:1		0.25±0.5	0.20±0.3	0.13	0.785546	-	-
**Triacylglycerides with indicated fatty acid + sum of two fatty acid residues**							
TG(14:0_40:5)		0.85±0.6	0.68±0.5	0.28	0.607151	-	-
TG(16:1_38:5)		0.80±1.1	2.06±1.8	0.76	0.243384	-	-
TG(17:1_36:5)		2.90±2.8	2.35±2.1	0.21	0.68713	-	*
TG(17:1_38:5)		0.35±0.5	0.38±0.5	0.06	0.906738	-	-
TG(17:2_34:3)		1.76±2.1	1.15±1.2	0.34	0.482322	-	-
TG(18:1_38:5)		0.43±0.3	0.89±0.6	0.90	0.191911	***	-
TG(18:2_38:5)		2.08±2.2	2.70±3.0	0.22	0.715206	-	-
TG(18:2_38:6)		1.21±1.2	1.91±2.3	0.34	0.593178	*	-
TG(18:3_35:2)		0.65±0.8	0.89±1.0	0.25	0.673756	-	-
TG(18:3_38:5)		1.40±1.6	1.84±1.5	0.26	0.640882	-	-
TG(20:0_32:4)		0.93±0.6	0.69±0.6	0.36	0.503649	-	-
TG(20:1_26:1)		1.31±1.4	1.02±0.9	0.23	0.649043	-	-
TG(20:1_32:3)		0.68±0.6	0.53±0.4	0.30	0.564843	*	*
TG(20:2_34:4)		0.58±0.4	0.54±0.3	0.12	0.821776	-	-
TG(20:2_36:5)		0.63±1.4	0.47±0.6	0.14	0.763023	-	-
TG(20:3_32:2)		0.74±0.3	0.50±0.2	0.80	0.147719	-	-
TG(20:3_34:3)		2.84±3.4	4.01±3.7	0.30	0.598217	-	-
TG(20:3_36:5)		0.59±0.5	0.46±0.4	0.30	0.564803	-	-
TG(20:4_32:0)		0.56±0.4	0.98±0.6	0.70	0.280495	-	-
TG(20:4_32:1)		2.17±1.7	4.55±2.1	1.14	0.087583	-	-
TG(20:4_32:2)		0.56±0.5	0.15±0.2	1.05	0.023952	***	*
TG(20:4_33:2)		1.98±1.7	2.57±2.8	0.23	0.708148	-	-
TG(20:4_34:0)		0.34±0.3	0.64±0.5	0.70	0.286327	*	-
TG(20:4_34:3)		12.96±12.6	35.19±39.8	0.68	0.329572	-	-
TG(20:4_35:3)		1.00±0.0	1.00±0.0	0.00	1	-	-
TG(20:4_36:4)		0.76±0.7	0.27±0.3	0.82	0.079548	-	-
TG(20:4_36:5)		0.86±0.5	0.87±1.1	0.01	0.983528	-	-
TG(22:0_32:4)		0.70±0.5	1.07±0.6	0.58	0.336584	-	-

TG(22:3_30:2)		0.89±0.8	1.45±1.0	0.57	0.350838	-	-
TG(22:4_32:2)		1.01±0.9	0.60±0.7	0.50	0.353595	-	-
TG(22:5_34:3)		0.40±0.4	0.51±0.3	0.28	0.586214	-	-
TG(22:6_34:2)		0.51±0.6	0.88±1.0	0.40	0.522042	-	-
TG(22:6_34:3)		1.10±1.2	0.74±0.7	0.35	0.472557	**	**
**Unesterified fatty acids**							
SCFA_C2	Acetic acid	1.64±0.5	2.00±0.6	0.63	0.299638	***	*
SCFA_C3	Propionic acid	1.36±0.5	1.26±0.2	0.22	0.610564	-	-
SCFA_C4	Butyric acid	0.87±0.5	0.59±0.1	0.73	0.093058	***	***
SCFA_C5	Pentanoic acid	0.39±0.1	0.39±0.1	0.07	0.906115	-	-
SCFA_C6	Hexanoic acid	0.65±0.4	0.46±0.1	0.68	0.119482	***	***
SCFA_C7	Heptanoic acid	0.74±0.1	0.98±0.3	0.94	0.184561	-	-
SCFA_C8	Octanoic acid	3.70±1.9	3.49±1.3	0.12	0.814372	**	**
FA(12:0)	Lauric acid	0.63±0.6	0.24±0.2	0.77	0.089951	***	***
FA(14:0)	Myristic acid	1.19±0.6	1.38±0.8	0.24	0.683387	-	*
FA(16:0)	Palmitic acid	1.46±1.3	1.52±1.4	0.04	0.940645	-	-
FA(18:0)	Stearic acid	0.97±0.4	0.83±0.2	0.38	0.430331	-	-
FA(18:1)	Octadecenoic acid	1.39±0.6	1.29±0.3	0.23	0.61955	-	-
FA(18:2)	Octadecadienoic acid	1.14±0.6	1.13±0.5	0.02	0.967049	-	-
FA(20:1)	Eicosenoic acid	0.18±0.3	0.75±0.7	0.94	0.182541	-	-
FA(20:3)	Eicosatrienoic acid	0.90±0.1	0.96±0.3	0.25	0.691114	-	-
Arachidonic acid	Arachidonic acid	1.50±0.5	0.85±0.2	1.57	0.001896	*	*
DHA	Docosahexaenoid acid	1.02±0.7	1.28±0.6	0.40	0.460406	-	-
EPA	Eicosapentaenoic acid	1.33±0.7	1.23±0.6	0.15	0.780627	-	-
DiCA(12:0)	Dodecanedioic acid	1.09±0.1	1.12±0.1	0.26	0.589476	*	-
DiCA(14:0)	Tetradecanedioic acid	0.99±0.2	1.08±0.3	0.32	0.604608	-	-
**Vitamins**							
B1	Thiamine	0.67±0.2	0.51±0.2	0.78	0.215709	*	*
B2	Riboflavin	0.36±0.3	0.45±0.3	0.25	0.662781	***	**
B5	Panthothenic acid	0.77±0.1	0.88±0.2	0.67	0.315604	*	*
Biotin	Biotin	0.97±0.3	0.92±0.2	0.20	0.703341	*	-
Choline	Choline	1.15±0.3	1.23±0.3	0.25	0.660919	-	-

Me-B12	Methylcobalamine	0.70±0.3	0.95±0.2	0.91	0.122008	-	-
Nicotinamide	Nicotinamide	0.07±0.2	0.19±0.4	0.38	0.555334	*	-
Nicotinic acid	Nicotinic acid	1.32±0.5	1.37±0.3	0.11	0.834057	-	*
Pyridoxal	Pyridoxal	2.24±0.8	2.44±0.4	0.32	0.500383	***	***
Pyridoxamine	Pyridoxamine	1.09±0.1	1.21±0.2	0.62	0.335521	-	-
Pyridoxine	Pyridoxine	1.12±0.1	1.18±0.2	0.31	0.631594	-	-
TPP	Thiamine pyrophosphate	1.24±0.2	1.25±0.2	0.04	0.942402	-	-
**Other metabolites**							
H1	Sum of Hexoses (mainly glucose)	0.26±0.3	0.10±0.1	0.64	0.179662	***	***
3-IAA	3-Indoleacetic acid	1.15±0.4	1.30±0.4	0.35	0.526682	-	-
3-IPA	3-Indolepropionic acid	0.98±0.2	0.77±0.2	0.97	0.090813	-	-
AconAcid	Aconitic acid	0.94±0.3	0.80±0.3	0.42	0.451194	*	-
Cortisol	Cortisol	0.84±0.3	1.04±0.7	0.30	0.639934	*	-
Cortisone	Cortisone	0.75±0.2	0.74±0.2	0.07	0.88994	-	-
DHEAS	Dehydroepiandrosterone sulfate	1.05±0.1	1.09±0.1	0.35	0.575671	-	-
HipAcid	Hippuric acid	0.77±0.9	0.77±0.7	0.00	0.995388	**	*
Hypoxanthine	Hypoxanthine	4.28±2.7	4.53±2.1	0.09	0.856256	*	-
Indole	Indole	0.63±0.3	0.66±0.2	0.10	0.84632	*	*
Ind-SO4	Indoxyl sulfate	1.06±0.4	0.93±0.2	0.40	0.374699	-	-
Lactate	Lactate	15.98±5.5	17.71±2.5	0.38	0.40771	***	***
OH-GlutAcid	3-Hydroxyglutaric acid	1.63±1.1	2.19±0.9	0.55	0.310096	***	*
p-Cresol-SO4	p-Cresol sulfate	0.73±0.3	0.56±0.0	0.83	0.060106	**	**
Suc	Succinic acid	0.97±0.2	0.78±0.1	1.08	0.024482	-	-
Trigonelline	Trigonelline	1.00±0.1	0.96±0.1	0.51	0.317939	-	-
Xanthine	Xanthine	0.62±0.3	0.39±0.1	0.94	0.049905	*	-

General linear model metabolite ~ age.group + bacterial.origin + fermentative.type was used

- p>0.05

* 0.05> p >0.01

** 0.01 > p > 0.001

*** p<0.001
