# Supplementary material for: Culture-based studies of intestinal lactobacilli in young people and centenarians
Source: Front Microbiol. 2026 Mar 10;17:1746411. doi: 10.3389/fmicb.2026.1746411 (PMC13008948; doi:10.3389/fmicb.2026.1746411)
Supplement: Supplementary file 3 [file Table_3.pdf]

Supplementary table 3. Biochemical profile of lactobacilli isolated from old and young people (API 50): ferment carbohydrates (yellow or green) or does not (purple).

species	old/young	Glycerol	Erythritol	D-Arabinose	L-Arabinose	D-Ribose	D-Xylose	L-Xylose	D-Adonitol	Methyl-βD-Xylopyranoside	D-Galactose	D-Glucose	D-Fructose	D-Mannose	L-Sorbose	L-Rhamnose	Dulcitol	Inositol	D-Mannitol	D-Sorbitol	Methyl-αD-Mannopyranoside	Methyl-αD-Glycopyranoside	N-Acetylglucosamine	Amygdalin	Arbutin	Esculin ferric citrate	Salicin	D-Cellobiose	D-Maltose	D-Lactose (bovine origin)	D-Melibiose	D-Saccharose (sucrose)	D-Trehalose	Inulin	D-Melezitose	D-Raffinose	Amidon (starh)	Glycogen	Xylitol	Gentiobiose	D-Turanose	D-Lyxose	D-Tagatose	D-Fucose	L-Fucose	D-Arabitol	L-Arabitol	potassium Gluconate	potassium 2-ketogluconate	potassium 5-ketogluconate
		1	2	3	4	5	6	7	8	9	10	11	12	13	14	15	16	17	18	19	20	21	22	23	24	25	26	27	28	29	30	31	32	33	34	##	##	##	##	##	##	##	##	##	##	##	##	##	##	##
Lactocaseibacillus rhamnosus	old	##	##	##	##	##	##	##	##	##	##	##	##	##	##	##	##	##	##	##	##	##	##	##	##	##	##	##	##	##	##	##	##	##	##	##	##	##	##	##	##	##	##	##	##	##	##	##	##	##
Lactocaseibacillus rhamnosus	old	##	##	##	##	##	##	##	##	##	##	##	##	##	##	##	##	##	##	##	##	##	##	##	##	##	##	##	##	##	##	##	##	##	##	##	##	##	##	##	##	##	##	##	##	##	##	##	##	##
Lactocaseibacillus rhamnosus	old	##	##	##	##	##	##	##	##	##	##	##	##	##	##	##	##	##	##	##	##	##	##	##	##	##	##	##	##	##	##	##	##	##	##	##	##	##	##	##	##	##	##	##	##	##	##	##	##	##
Lactocaseibacillus rhamnosus	young	##	##	##	##	##	##	##	##	##	##	##	##	##	##	##	##	##	##	##	##	##	##	##	##	##	##	##	##	##	##	##	##	##	##	##	##	##	##	##	##	##	##	##	##	##	##	##	##	##
Lactocaseibacillus rhamnosus	young	##	##	##	##	##	##	##	##	##	##	##	##	##	##	##	##	##	##	##	##	##	##	##	##	##	##	##	##	##	##	##	##	##	##	##	##	##	##	##	##	##	##	##	##	##	##	##	##	##
Lactocaseibacillus rhamnosus	old	##	##	##	##	##	##	##	##	##	##	##	##	##	##	##	##	##	##	##	##	##	##	##	##	##	##	##	##	##	##	##	##	##	##	##	##	##	##	##	##	##	##	##	##	##	##	##	##	##
Lacticaseibacillus paracasei	old	##	##	##	##	##	##	##	##	##	##	##	##	##	##	##	##	##	##	##	##	##	##	##	##	##	##	##	##	##	##	##	##	##	##	##	##	##	##	##	##	##	##	##	##	##	##	##	##	##
Lacticaseibacillus paracasei	young	##	##	##	##	##	##	##	##	##	##	##	##	##	##	##	##	##	##	##	##	##	##	##	##	##	##	##	##	##	##	##	##	##	##	##	##	##	##	##	##	##	##	##	##	##	##	##	##	##
Lacticaseibacillus paracasei	young	##	##	##	##	##	##	##	##	##	##	##	##	##	##	##	##	##	##	##	##	##	##	##	##	##	##	##	##	##	##	##	##	##	##	##	##	##	##	##	##	##	##	##	##	##	##	##	##	##
Lactobacillus curvatus (Latilactobacillus curvatus)	young	##	##	##	##	##	##	##	##	##	##	##	##	##	##	##	##	##	##	##	##	##	##	##	##	##	##	##	##	##	##	##	##	##	##	##	##	##	##	##	##	##	##	##	##	##	##	##	##	##
Lactobacillus harbinensis (Schleiferilactobacillus harbensis)	old	##	##	##	##	##	##	##	##	##	##	##	##	##	##	##	##	##	##	##	##	##	##	##	##	##	##	##	##	##	##	##	##	##	##	##	##	##	##	##	##	##	##	##	##	##	##	##	##	##
Lactobacillus sakei (Latilactobacillus sakei)	old	##	##	##	##	##	##	##	##	o	##	##	##	##	##	##	##	##	##	##	##	##	##	##	##	##	##	##	##	##	##	##	##	##	##	##	##	##	##	##	##	##	##	##	##	##	##	##	##	##
Lactobacillus gasseri	old	##	##	##	##	##	##	##	##	##	##	##	##	##	##	##	##	##	##	##	##	##	##	##	##	##	##	##	##	##	##	##	##	##	##	##	##	##	##	##	##	##	##	##	##	##	##	##	##	##
Lactobacillus gasseri	old	##	##	##	##	##	##	##	##	##	##	##	##	##	##	##	##	##	##	##	##	##	##	##	##	##	##	##	##	##	##	##	##	##	##	##	##	##	##	##	##	##	##	##	##	##	##	##	##	##
Lactobacillus gasseri	old	##	##	##	##	##	##	##	##	##	##	##	##	##	##	##	##	##	##	##	##	##	##	##	##	##	##	##	##	##	##	##	##	##	##	##	##	##	##	##	##	##	##	##	##	##	##	##	##	##
Limosilactobacillus frumenti	old	##	##	##	##	##	##	##	##	##	##	##	##	##	##	##	##	##	##	##	##	##	##	##	##	##	##	##	##	##	##	##	##	##	##	##	##	##	##	##	##	##	##	##	##	##	##	##	##	##
Limosilactobacillus frumenti	old	##	##	##	##	##	##	##	##	##	##	##	##	##	##	##	##	##	##	##	##	##	##	##	##	##	##	##	##	##	##	##	##	##	##	##	##	##	##	##	##	##	##	##	##	##	##	##	##	##
Lentilactobacillus otakiensis	old	##	##	##	##	##	##	##	##	##	##	##	##	##	##	##	##	##	##	##	##	##	##	##	##	##	##	##	##	##	##	##	##	##	##	##	##	##	##	##	##	##	##	##	##	##	##	##	##	##
